# The Role of Mindful Parenting in Individual and Social Decision-Making in Children

**DOI:** 10.3389/fpsyg.2019.00550

**Published:** 2019-03-20

**Authors:** Kristyn Wong, Laurel M. Hicks, Terri G. Seuntjens, Christopher J. Trentacosta, Tessel H. G. Hendriksen, Marcel Zeelenberg, Marion I. van den Heuvel

**Affiliations:** ^1^ Department of Psychiatry and Human Behavior, Alpert Medical School of Brown University, Providence, RI, United States; ^2^ Bradley Research Center, E. P. Bradley Hospital, Providence, RI, United States; ^3^ Department of Psychology, University of Denver, Denver, CO, United States; ^4^ Independent Researcher, Tilburg, Netherlands; ^5^ Department of Psychology, Wayne State University, Detroit, MI, United States; ^6^ Faculty of Social and Behavioral Sciences, Tilburg University, Tilburg, Netherlands; ^7^ Department of Social Psychology, Tilburg University, Tilburg, Netherlands; ^8^ Department of Marketing, VU Amsterdam, Amsterdam, Netherlands; ^9^ Department of Cognitive Neuropsychology, Tilburg University, Tilburg, Netherlands

**Keywords:** mindful parenting, children, choice-related stress, decision-making, sharing

## Abstract

Children are confronted with an increasing amount of choices every day, which can be stressful. Decision-making skills may be one of the most important “21st century skills” that children need to master to ensure success. Many aspects of decision-making, such as emotion regulation during stressful situations, develop in the context of caregiver-child interactions. This study examined whether mindful parenting predicts children’s individual and social decision-making. The current study included 63 mother-child dyads from The Netherlands (Child *M_age_* = 5.11, *SD* = 0.88, 50.8% girls). Mothers completed the Dutch version of the Interpersonal Mindfulness in Parenting Scale (IM-P). A “Choice Task” was developed to measure individual decision-making skills, and a “Sharing Task” was created to measure social decision-making in young children. Higher maternal mindful parenting significantly predicted more sharing after controlling for covariates (child age, sex, SES, maternal education level; *Wald* = 4.505, *p* = 0.034). No main effect of maternal mindful parenting was found for any of the individual decision-making measures. These findings suggest that mindful parenting supports children’s social decision-making. Future research should investigate if the combination of mindful parenting and children’s early decision-making skills predict key developmental outcomes.

Rapid societal advancements in daily life and the modern economy have led to demand for the next generation to develop a multitude of skills beyond traditional academic learning to prepare them for the “real world” (Binkley et al., 2012). Scholars and professionals alike stress the importance of these learning, adaptation, and interpersonal skills, often referred to as “21st century skills.” Decision-making skills may be one of the most essential 21st century skills for children to master as they play an important role in making effective and informed choices as children navigate real-life problems as they arise. It is critically important for children to learn to make effective decisions and to successfully manage their emotions and behaviors as they deal with the consequences of those decisions.

Decision-making often occurs in affectively charged contexts (Schwarz, 2000), which means that decision-making requires adaptive regulation of emotions. Moreover, a large body of literature provides evidence that emotional self-regulation regulation develops within the context of high-quality parent-child relationships (Thompson, 1994), beginning in the early years of life (Beeghly and Tronick, 2011). Furthermore, the quality of mother-child interactions has been found to be associated with the children’s development of adaptive social behaviors including positive emotion expression and assertiveness (Denham et al., 1991). In a longitudinal study examining mother-child interaction and child outcomes, maternal parenting behavior characterized as sensitive and supportive in early childhood was linked to later child academic performance and social behavior in middle childhood (Morrison et al., 2003).

Mindful parenting has been identified as a promising approach that promotes emotion regulation in parents and children (Duncan et al., 2009). Thus, one way to support children’s development of 21st century sills may be to support parents’ use of mindful parenting. Mindful parenting is rooted in the construct of mindfulness, which Kabat-Zinn (2003) conceptualizes as the practice of awareness in the moment that is cultivated by increased attention without judgment and reactivity. When applied to the context of the parent-child relationship, mindful parenting is posited to not only improve parent emotion regulation but also to foster healthy parent-child relationships and promote improved child emotion regulation (Duncan et al., 2009). However, whether mindful parenting could also be advantageous for a child’s specific 21st century skills that involve emotion regulation, such as social decision-making behaviors, is still unknown.

## Decision-Making

Empirical studies that examine decision-making in children focus on multiple dimensions of self-regulation, including inhibitory control and the delay of gratification (Kerr and Zelazo, 2004; Geurts et al., 2006; Kidd et al., 2013; Lee and Carlson, 2015). These studies primarily use paradigms such as the Marshmallow Task (Mischel, 1974, 2014) and adapted versions of the Iowa Gambling Task (Bechara et al., 1994; Kerr and Zelazo, 2004) that require children to make choices about rewards that have immediate or delayed gratification (i.e., receiving a specific amount of a prize). Kerr and Zelazo (2004) examined affective decision making in preschool children using an Iowa Gambling Task for children. Age differences were found between 3- and 4-year-old preschoolers such that 4-year-old children made more advantageous choices across trials compared to 3-year-old children. Crone and van der Molen (2004) examined children’s decision-making in a larger age range of children (8–18 years old) and found evidence suggesting that as children aged their awareness of future consequences increased. Furthermore, the youngest group of children evidenced failure of the ability to anticipate future outcomes. Together, evidence from these studies suggest that older children are more likely to demonstrate the ability to delay rewards and make more advantageous decisions than younger children, which may be attributed to differences in brain maturation.

Developmental psychologists have a long-standing interest in the development of prosocial behavior (Eisenberg et al., 1983, 2015), including the notion that parenting behaviors may influence children’s prosocial behaviors in early childhood. Sensitive parents could socialize and model prosocial behaviors for children which, in turn, might influence the development of these behaviors in their children. Alternatively, others suggest that sensitive parents are better able to facilitate children’s development of prosocial behaviors by fostering children’s awareness of others’ needs (for a review, see Eisenberg and Valiente, 2002). Newton et al. (2014) examined associations between school-aged children’s parent reported prosocial behavior and observed parent sensitivity using a large longitudinal dataset and found support for a bi-directional relationship between children’s prosocial behaviors and maternal sensitivity (but not for paternal sensitivity).

Gender differences have also been examined in relation to children’s prosocial behavior, with the findings favoring girls as demonstrating increased sharing behavior and social competence (Burford et al., 1996; Fabes et al., 1999), which might be driven by differences in parental gender socialization. Examining children’s prosocial behavior within a forced-choice laboratory paradigm may provide more insight into children’s social decision-making and an opportunity to further explore gender effects. Although a few studies have examined children’s social decision-making within the context of manipulated social environments (Prencipe and Zelazo, 2005; Leimgruber et al., 2012; Weller and Lagattuta, 2014), to our knowledge, none have examined children’s social decision-making in relation to parenting. Exploring parenting as a possible correlate or predictor of children’s social decision-making behavior is important because parents play a critical role in children’s ability to regulate attention and emotion, which are key aspects of decision-making.

## Stress, Mindfulness, and Decision-Making

Extant research reports that stress negatively affects physical and emotion wellbeing in a wide range of contexts and suggests that stress and decision-making are intertwined. Specifically, stress negatively affects decision-making through its impacts on the underlying neural mechanisms of decision-making (Preston et al., 2007; for review Starcke and Brand, 2012). Findings from this work suggest bidirectional relationships between stress and decision-making, where stress can negatively impact decision-making behavior, but also that specific decisions can elicit a stress response (Wemm and Wulfert, 2017). Much of the research examining the impacts of stress on decision-making has been conducted in adult populations, with a few studies exploring how contextual stress impacts adolescent decision making. Although some empirical work suggests that, among adolescents, increased stress is associated with risky decision making (Galvan and McGlennen, 2011; Johnson et al., 2012), to our knowledge there has been no work examining how stress influences either individual or social decision-making in early childhood.

Individual stress management strategies, such as being mindful in stressful situations, can lessen the effects of stress on physiology and emotion and may be a key factor in supporting decision-making. In childhood, the parent–child relationship plays a significant role in promoting children’s optimal development (Hartup, 1989). Parents often help to regulate the child’s emotion, especially in times of stress (Haley and Stansbury, 2003). Warm, consistent caregiving relationships provide the ideal environment for children to develop and refine their emotion regulation capacities and social emotional competence (Cassidy, 1994; Thompson, 1994). Therefore, examining how parenting contributes to the development of children’s decision-making skills in early childhood may provide insight into the most effective ways parents can facilitate children’s development of critical thinking and problem-solving skills related to decision-making in both individual and social contexts.

## Mindful Parenting

Empirical studies suggest that a number of neural and cognitive mechanisms influence the development of decision-making skills; additionally, high-quality relationships with caregivers may further facilitate the development of these skills. Early in life, interactions with parents provide external regulation of emotion and over time children develop independent regulatory capacities that should facilitate decision-making (Thompson and Meyer, 2007). Parents’ own ability to control their emotions influences their interactions with their children, and variations in parent emotion regulation are determined by a combination of cognitive, social, physiological, and neurobiological factors (Morris et al., 2007). Parents who are unable to model successful regulation through behavior and parenting practices contribute to emotion regulation difficulties in their children (Rutherford et al., 2015), which may indirectly impact the development of children’s decision-making abilities. One promising construct, mindful parenting (Kabat-Zinn and Kabat-Zinn, 1997; Duncan et al., 2009), is posited to underlie parents’ own emotion regulation, and thus may be an important predictor of children’s emotion regulation and decision-making.

Originally proposed by Kabat-Zinn and Kabat-Zinn (1997), mindful parenting is conceptualized as the practice of being present and aware in everyday interactions with children through paying attention without judgment as each moment unfolds. Duncan et al. (2009) integrated and extended the model of mindful parenting by incorporating the principles of classic mindfulness theory as applied to parent-child relationships. The model includes five dimensions specific to parent-child interactions; (1) listening with full attention, (2) non-judgmental acceptance of self and child, (3) emotional awareness of self and child, (4) self-regulation in the parenting relationship, and (5) compassion for self and child. These dimensions approach parenting in a way that facilitates being present in daily interactions, parenting more calmly, and engaging in increased emotion regulation. As a result, parenting behavior becomes more consistent and responsive over time and parent-child relationships become characterized as more positive, warm, and supportive and filled with less negativity, conflict, and judgment.

Dispositional mindfulness is thought to be a natural way of being mindful in day-to-day life and is associated with improved emotion regulation (Fogarty et al., 2013), improved mental health symptoms (Bravo et al., 2018; Hicks et al., 2018), and reduced stress (Bergin and Pakenham, 2016). There is growing interest in examining applications of mindfulness in children and parents (Thompson and Gauntlett-Gilbert, 2008; Coatsworth et al., 2010; Semple and Lee, 2014). Much of the existing work examines the role of mindful parenting in adolescent-parent relationships (Duncan, 2007; Geurtzen et al., 2015; Lippold et al., 2015); however, very few studies have examined mindful parenting in parents of younger children (Srivastava et al., 2011; Laurent et al., 2017). Laurent et al. (2017) examined whether mindful parenting was related to both mother and infant physiological responses to stress in the Still Face Paradigm. Results suggested that only mindful parenting (not dispositional mindfulness) was associated with faster cortisol recovery after the stressor for mothers. No significant main effects were identified for mindful parenting on infants’ cortisol responses. In a sample of children aged 3–17 years old, an indirect relationship between parent dispositional mindfulness and child internalizing and externalizing problems through mindful parenting and negative parenting practices was found (Parent et al., 2016). This finding aligns with other work suggesting that although both mindful parenting and dispositional mindfulness are positively correlated, only mindful parenting is found to be associated with parenting related constructs like parenting stress. On the other hand, dispositional mindfulness is more closely associated with broader aspects of parents’ mental health (Corthorn and Milicic, 2016). Mindful parenting, particularly in early childhood, may enable parents to provide consistent and positive caregiving, which provides the foundation needed to facilitate children’s emotion regulation and decision-making skills.

## Current Study

In the current study, we explored the role of mindful parenting in fostering individual and social decision-making. For the sake of clarity, we use the term “individual decision-making” when the consequences of the decision are only for the individual making it, whereas we use the term “social decision-making” when the consequences also impact another individual (e.g., a friend or stranger), with “prosocial” referring specifically to positive forms of social decision-making behaviors. This study was the first to explore the association between self-reported maternal mindful parenting and observed decision-making behavior in children. First, we examined whether mindful parenting was associated with children’s individual decision-making behavior. We hypothesized that children with more mindful mothers would exhibit less observed stress, doubt, and confirmation seeking in a choice task. Second, we hypothesized that maternal mindful parenting would predict children’s level of social decision-making behavior in a laboratory administered sharing task. Additionally, age and sex differences in these associations were explored. Given the aforementioned gender differences in prosocial behavior favoring girls, we anticipated that girls would display higher levels of social decision-making. However, we did not have specific hypotheses regarding the extent to which mindful parenting would impact decision-making for girls related to boys.

## Materials and Methods

### Participants and Procedure

Mothers and children were recruited from a community database of interested parents and *via* Facebook advertisements, and were invited to participate in a behavioral study examining child decision-making and sharing (“Choosing & Sharing in Young Children”). A total of 64 mother-child dyads participated in the study. For the purpose of the current study, only those mother-child dyads were included that had usable questionnaire and behavioral data. We excluded one dyad with missing data on the mindful parenting questionnaire, resulting in a total sample of 63 4-to-6-year-olds (child *M_age_* = 5.11, *SD* = 0.88; 32 girls, 31 boys). Detailed sample characteristics are shown in Table 1.

**Table 1:** Sample characteristics.

Outcome	Value
Children’s age, years	5.11 ± 0.88
**Child sex, %**	
Female	50.8
Male	49.2
**Number of siblings, %**	
0	9.5
1	63.5
2	20.6
3 or more	6.4
**Children sticker sharing group, %**	
Sharing	39.7
Non-sharing	60.3
Mindful parenting score	117.89 ± 9.91
**Education parents, %**	
General vocational training	14.3
Higher vocational training	52.4
University degree	25.4
Missing	7.9

Values presented as mean ± SD where appropriate.

For this project, mother-child dyads were invited to the Tilburg University Life Span Lab. Mothers filled out questionnaires regarding their own and their child’s behavior, and children participated in several behavioral tasks. In the current research, we focus on the association between mother-reported mindful parenting and the child’s behavior on an individual decision-making task (“Choice Task”) and a social decision-making task (“Sharing Task”). All children started with a playful game that involved blowing bubbles to make them feel comfortable before starting the experiment. During the study, at least two researchers were present: a test leader who completed the behavioral tasks with the child, and a second researcher who operated the cameras and filled out an observation form. If any siblings came along, there was a third researcher who played with the siblings. In several families, two or more siblings participated separately in the experimental procedure (13 families participated with two children, one family participated with three children). In this case, the third researcher made sure the siblings did not observe each other performing the tasks. The full procedure was recorded with three cameras from different angles and lasted about 60 min in total. The study was approved by the ethical review board of Tilburg University and was conducted in full compliance with the Helsinki declaration. All included mothers (and fathers) provided informed consent before participating.

### Measures

#### Maternal Mindful Parenting

The Interpersonal Mindfulness in Parenting Scale Dutch Version (IM-P; Duncan, 2007; de Bruin et al., 2014) was used to measure maternal mindful parenting. The 31-item IM-P assesses five dimensions of mindful parenting: listening with full attention (e.g., “I find myself listening to my child with one ear because I am busy doing or thinking about something else at the same time”), emotional awareness of self and child (e.g., “I notice how changes in my child’s mood affect my mood”), self-regulation in parenting relations (e.g., “When I’m upset with my child, I notice how I am feeling before I take action”), non-judgmental acceptance of self and child (e.g., “I listen carefully to my child’s ideas, even when I disagree with them”), and compassion for self and child (e.g., “I tend to be hard on myself when I make mistakes as a parent”). An IM-P total score can also be computed from 29 items (excluding two items), as a global indicator of mindful parenting (Duncan, 2007). Items are measured on 5-point scales ranging from 1, *never true*, to 5, *always true*. Because of the low reliability of the subscales, only the IM-P total score was used in study analyses (*α* = 0.86). Since parenting practices can differ between children, mothers completed the questionnaire separately for each child if more than one child participated in the study.

#### Choice Task

**Figure 1:**
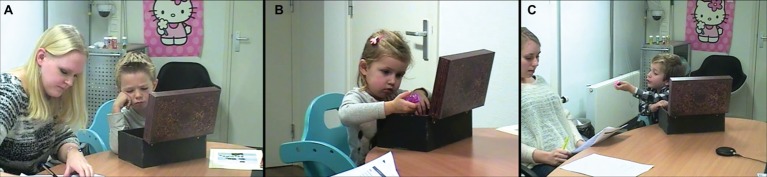
Snapshots of Choice Task setup (individual decision-making). Snapshots illustrate examples of children who scored high on decision-related stress **(A)**, doubt/indecisiveness **(B)**, and confirmation seeking **(C)**.

To measure individual decision-making in young children, we developed a task in which children chose from a large assortment of toys under medium pressure. This choice task took place at the end of the study. The children were told they earned a small gift. They were then presented with a wooden treasure chest full of toys (45 total toys: 15 boy toys, 15 girl toys, 15 gender-neutral toys). They were then instructed to open the chest, pick a toy they like the best, and then to close the chest. To induce some choice pressure, it was emphasized that when the child closes the box, they could not change presents anymore. See Figure 1 for snapshots of the choice task.

A coding system was developed by the authors (Supplemental Material S1), based on observations of children performing the choice task. Child behaviors during the choice as were rated based on the coding system by a trained research assistant after the study, using the recordings. The following constructs were coded from the videos: time to decision making, decision-related stress, doubt/indecisiveness, and confirmation seeking. Duration of choosing (in seconds) was measured by timing the number of seconds between opening and closing the chest with a stopwatch. The interclass correlation (ICC) was computed by recoding 25% of the videos by a second rater. In this article we adhere to Cicchetti’s (1994) guidelines for interpreting ICC.

To assess decision-related stress, videos were coded using a six point scale, ranging from zero to five points: 0 = no signs of stress, 1 = shows light stress (e.g., twisting on the chair), 2 = shows moderate stress (e.g., fingers in mouth, restlessness, light frown), 3 = shows clear stress (e.g., clear restlessness, frowning, tense posture), 4 = shows high stress (e.g., very tense, strong frowning, child indicates to have a hard time), and 5 = shows extreme stress (e.g., contorted face, crying, blushing). The ICC was *r* = 0.76, indicating excellent inter-rater reliability.

To assess doubt/indecisiveness, videos were coded using a six point scale, ranging from zero to five points: 0 = seems to experience no doubt (e.g., directly takes what he/she wants), 1 = seems to experience light doubt (e.g., holds different options in hand, but chooses without trouble), 2 = seems to experience moderate doubt (e.g., holds different options in hand, hesitates and then chooses), 3 = seems to experience clear doubt (e.g., holds different options in hand, puts it back and picks it back up, clearly hesitating), 4 = seems to experience a lot of doubt (e.g., holds numerous options in hand and puts them back or clearly hesitates between two or more options, picks the options back up that were put down, reconsiders almost made decisions, child indicates that he/she is in doubt), and 5 = seems to experience extreme doubt (e.g., persisting doubt, keeps hesitating and keeps reconsidering made decisions). The ICC was *r* = 0.89, indicating excellent inter-rater reliability.

To assess confirmation seeking, videos were coded using a six point scale, ranging from zero to five points: 0 = does not ask for help and does not seem to need it, 1 = lightly asks without talking/not directly, for help (e.g., by looking at the test leader), 2 = asks without talking/not directly for help (e.g., thinking out loud, seeking eye-contact with the test leader), 3 = carefully and directly asks for help (e.g., asking what the test leader prefers), 4 = clear and directly asking for help (e.g., asks if the mother can come to help), and 5 = strongly asks for help (e.g., asks the mother/test leader to choose for them). The ICC was *r* = 0.84, indicating excellent inter-rater reliability. Two cases had to be excluded for the constructs decision-related stress, doubt/indecisiveness, and confirmation seeking due to child compliance problems. More specifically, one child had to go to the bathroom, and a second child could not pick a toy and started crying. Since the latter scenario may be an extreme case of decision-making disturbance, we included this second case for all subscales but coded him as missing for time.

#### Sharing Task

**Figure 2:**
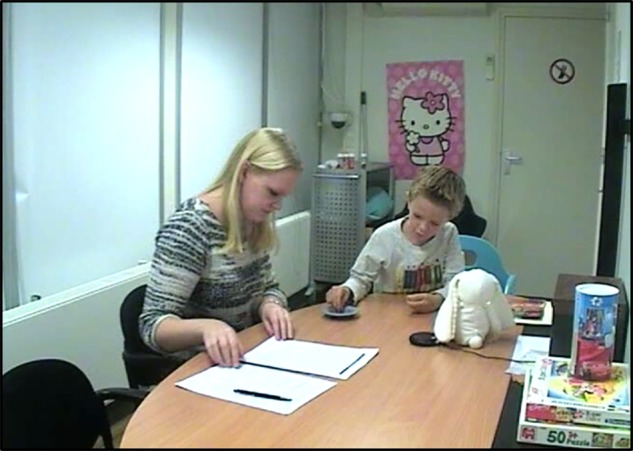
Snapshot of Sharing Task setup (social decision-making). The child was first introduced to a stuffed animal (“Konijn,” Dutch for Bunny). Konijn has five stickers that he wants to share with the child. Subsequently, the child is asked to decide how many stickers Konijn gets and how many he/she want to keep. Two small trays were placed in front of the child, one for Konijn and one for the child.

To measure child social decision-making (sharing behavior), we used a sharing task in which children had to decide how many stickers to share with a stuffed animal. We adapted the sharing task from Chernyak and Kushnir (2013) and Paulus and Moore (2014). First, the experimenter introduced the child to a stuffed animal (“Konijn,” Dutch for Bunny). Then the experimenter took two small trays and gave one of the trays to Konijn and the other to the child. Participants were then told that Konijn was a sweet bunny and that he had five stickers that he wanted to share with the child. Children then received the five stickers and were told that they could decide how many of the stickers they wanted to share with Konijn by placing the stickers either in Konijn’s tray or in their own tray. The number of stickers in Konijn’s tray was used as measure for sharing. Before the experiment started, children were asked to point to their own tray and to Konijn’s tray as a final check. We used five stickers to force children to create an uneven distribution [i.e., either to prioritize themselves or to prioritize Konijn, Chernyak and Kushnir (2013) used three stickers instead of five]. Children were divided in two groups: a “high-sharing” group (majority of stickers given to Konijn – usually 3) and “low-sharing” group (majority of stickers kept to themselves – usually 2 given to Konijn and occasionally only 1). See Figure 2 for a snapshot of the sharing task.

#### Covariates

Several covariates were measured and included into subsequent statistical models, including age and sex of the child, socioeconomic status (SES) of the family, and education level of the mother. Mothers reported these via a questionnaire. SES was assessed via averaging responses (1 = never, 3 = always) to the following three items: “In the past year, did you have problems at the end of the month paying your fixed costs (for example, rent, groceries, and utilities)?”, “In the past year, did you worry about your financial situation?”, and “In the past year, did you have to borrow money from friends or family?”. SES was examined as a covariate because children from households with lower SES may struggle with developmental competences, including emotion regulation and decision-making, due to the experience of poverty-related stressors (Raver, 2004).

### Statistical Approach

First, inter-correlations between the different measures of decision-making in the Choice Task were computed with Pearson’s correlations. Next, we examined whether maternal mindful parenting was associated with measures of individual decision-making (i.e., time, decision-related stress, doubt/indecisiveness, and confirmation seeking) by running four hierarchical regression models, one model per construct. The first step of the hierarchical regression model contained the maternal mindful parenting score. In the next step, the covariates (i.e., child age, child sex, number of siblings, SES, and maternal education level) were added. The last step contained a sex interaction. Significant sex interactions were followed up with simple effects tests.

To examine the association between maternal mindful parenting and child sharing behavior, we categorized children into groups based on their sharing behavior. Because most children either gave two or three stickers to Konijn (only one child gave one sticker, none of the children gave four or more stickers), we computed a binary variable for sharing. Subsequently, we ran a hierarchical (binary) logistic regression model with the same steps as the above described hierarchical regression model, but with sharing group as the dependent variable. All analyses were performed in SPSS Statistics 22 (IBM, 2013), and a *p* threshold of 0.05 was used for significance testing.

## Results

### Child Individual Decision-Making

The four measures (i.e., time, decision-related stress, doubt/indecisiveness, and confirmation seeking) showed high, positive inter-correlations (Table 2). We found no sex differences on any measures of individual decision-making. Strong effects were found for child’s age for time (*r* = 0.30, *p* = 0.021), decision-related stress (*r* = 0.38, *p* = 0.003), and doubt/indecisiveness (*r* = 0.40, *p* = 0.002), indicating that older children experienced more difficulty with this individual decision-making process than younger children.

**Table 2:** The inter-correlations between the four measures of the choice task: time, decision-related stress, doubt/indecisiveness, and confirmation seeking.

	Time (s)	Decision-related stress	Doubt/indecisiveness	Confirmation seeking
Time (s)	–	0.76^*^	0.82^*^	0.60^*^	
Decision-related stress		–	0.71^*^	0.43^*^	
Doubt/indecisiveness			–	0.65^*^	
Confirmation seeking				–	

* Correlation is significant at the *p* < 0.01 level.

No main effect of mindful parenting was found for any of the individual decision-making measures (time: *F* = −0.554, *p* = 0.582; decision-related stress: *F* = −0.949, *p* = 0.347; doubt/indecisiveness: *F* = −0.703, *p* = 0.485; confirmation seeking: *F* = −1.103, *p* = 0.275). Effects remained non-significant after controlling for covariates. We did find trending interactions with sex for doubt/indecisiveness (*t* = 1.95, *p* = 0.057) and confirmation seeking (*t* = −2.06, *p* = 0.058). However, when controlling for covariates, these interactions became non-significant. No significant interactions with child age were found for any of the constructs (time: *F* = 0.442, *p* = 0.661; decision-related stress: *F* = 0.594, *p* = 0.555; doubt/indecisiveness: *F* = 0.469, *p* = 0.641; confirmation seeking: *F* = 1.101, *p* = 0.276).

### Child Social Decision-Making

In the current sample, most children were included in the “low-sharing” group (*n* = 38, 60.3%) versus the “high-sharing” group (*n* = 25, 39.7%). We found no sex differences in sharing group membership (χ*^2^* = 0.21, *p* = 0.648), and children in the “low-sharing” group did not significantly differ in age from those in the “high-sharing” group (*t* = −1.47, *p* = 0.147).

Maternal mindful parenting significantly predicted sharing behavior [*Wald* = 4.82, *p* = 0.028; Exp(B) = 1.067 (95% CI = 1.007–1.131)]. Even after controlling for covariates (child age, sex, SES, maternal education level) the association remained significant [*Wald* = 4.51, *p* = 0.034; Exp(B) = 1.066 (95% CI = 1.005–1.131)]. Children who were exposed to higher levels of maternal mindful parenting shared significantly more stickers with the stuffed animal than children exposed to lower levels of maternal mindful parenting. We did not find significant sex (*Wald* = 0.247, *p* = 0.619) or age (*Wald* = 0.281, *p* = 0.596) interactions.

## Discussion

The purpose of the current study was to investigate associations between maternal mindful parenting and preschool children’s individual and social decision-making. Although the empirical research concerning mindful parenting is growing, few studies have specifically focused on mindful parenting and associations with preschool children’s development. To the best of our knowledge, this study is the first to test associations between mindful parenting and young children’s decision-making behavior.

### Mindful Parenting and Individual Decision-Making

Primary analyses examining mindful parenting and behavioral indicators of children’s decision-making behavior indicated no significant main effects for maternal mindful parenting on children’s time to decision-making or on children’s decision-related stress, doubt, and confirmation seeking. One possible explanation for the null findings in the current study related to mindful parenting and individual decision making is that the individual decision-making choice task and the behaviors coded from this task may not have adequately tapped into processes that would be influenced by the parent–child relationship broadly or mindful parenting specifically. Another possibility is that individual decision-making is not as strongly related to mindful parenting because other aspects of children’s social context and individual differences in children’s temperament and personality play more important roles in fostering choice-related individual decision-making.

Notably, correlations between study variables of interest identified strong child age effects on children’s decision-making time, decision-related stress, and doubt/indecisiveness. Older children spent more time deciding between choices on the laboratory choice task and exhibited greater decision-related stress and doubt compared to younger children. Since analyses were only correlational, it is difficult to draw causal inferences for why this may be; however, one possible explanation for this association could be that older children’s cognitive capacities are more developed allowing them to think critically about the risks and benefits for choosing one toy over another. Additionally, taking time to examine each possible choice may have also led to delayed time to decision-making as well as doubtfulness. Future studies with larger samples should investigate if there is an interaction between decision-making, child age and mindful parenting. More specifically, research across a broader age range could examine whether there are differential effects of mindful parenting on individual decision-making behavior in younger versus older children.

### Mindful Parenting and Social Decision-Making

Next, we examined the relationship between mindful parenting and children’s social decision-making. Maternal mindful parenting predicted child prosocial decision-making behavior during a sharing task even after controlling for demographic factors such as child age, sex, SES, and maternal education level. Children with more mindful mothers were more likely to engage in sharing behavior than those with mothers who were low in mindful parenting, which is noteworthy because prosocial sharing behavior is a normative developmental attainment of the preschool years (Paulus and Moore, 2014). Therefore, identifying a contextual factor that seems to influence the extent to which young children share with others suggests that programs to promote parents’ mindful parenting could provide tangible benefits to young children’s early social development. Related to this idea, one possible explanation for the observed association between mindful parenting and social decision-making is that parents higher in mindful parenting model and emphasize prosocial behaviors in daily interactions with children. Another possibility is that mindful parenting contributes to parental positivity and less punitive discipline, both of which have been found to be associated with increased prosocial behaviors in young children (Knafo and Plomin, 2006). Future research should investigate whether there are specific domains from Duncan et al.’s (2009) model of mindful parenting that are more predictive of children’s prosocial decision-making than others. Unfortunately, this research question was not pursued in the current study due to low internal reliability of mindful parenting subscales.

### Study Limitations, Future Directions, and Implications

The current study is not without a few important limitations. First, the cross-sectional design means that we cannot draw firm causal inferences about the direct of effects in analyses of mindful parenting and children’s decision-making. Future studies could investigate changes in child decision-making after a mindful parenting intervention, which would increase understanding of the extent to which mindful parenting precedes and facilitates child decision-making.

Second, the current study included preschool aged children ranging from ages 4 to 6 years old; this broad age range may have contributed to some of the variability found in both the individual decision-making and social decision-making tasks. Future studies should consider comparisons between specific age groups to better understand how developmental differences across early childhood may be associated with decision-making.

Third, methodological limitations may have contributed to the pattern of findings. From a methodological perspective, our null finding related to individual decision-making may be related to the choice task used to assess individual decision-making. Because the choice task was newly developed for this study, it may be that the paradigm taps into multiple constructs including both individual decision-making as well as other related constructs such as impulsivity. Also, the use of self-report measures to assess mindful parenting introduces the possibility that results may be impacted by rater bias. Preliminary work has explored using observational ratings of parent-child interaction concurrently with self-reported mindful parenting (Duncan et al., 2015), which may be a useful methodological approach for future studies to corroborate self-reports of mindful parenting.

Fourth, this study did not consider additional parenting factors and individual child characteristics that may play an important role in the development of decision-making. Therefore, future studies should assess mindful parenting in concordance with other parenting constructs such as warmth and hostility as well as developmental assessments of children’s executive function, inhibitory control, and other early competencies that could promote decision-making. Furthermore, only the mindful parenting of the mother is considered in this study. Some research posits that fathers (or other co-parents) impart a specific and important effect on child development, including prosocial behavior (Gryczkowski et al., 2018). Therefore, future studies should include co-parents to further understand the role of the family context in promoting children’s decision-making skills.

Fifth, the study’s small sample size puts limitations on power and the types of analyses that could be used to test the research questions. Furthermore, the homogenous sample, which may have been compounded by including siblings from 14 families, limits the generalizability of the current study findings and highlights the need to include more diverse samples as people within different cultures may value decision-making differently. Finally, participants self-selected into the study which may increase bias due to potential increased interest in the subject matter compared to the general population.

As stress and emotional regulation are believed to contribute to the ability to make decisions, future studies should take a biopsychosocial approach when addressing these research questions. For example, one may consider actual physiological stress responses using measurements of cortisol or respiratory sinus arrhythmia throughout the choice and sharing tasks. In addition, considering contextual factors that may play a role in the stress response, such as environmental chaos (Lepore et al., 2010), may provide important insights into socio-ecological risk factors that could contribute to poor decision making.

In summary, findings from the current study suggest that mindful parenting may play an important role in the development of children’s prosocial decision-making. Future research should investigate how this association unfolds over time while also examining developmental outcomes associated with prosocial decision-making during early childhood. For example, early prosocial decision-making could foster successful adaptation across the school transition, which fits with evidence that social competence is a key indicator of school adjustment (Curby et al., 2008). Our findings also add to the growing evidence that parents’ use of mindfulness during caregiving should be incorporated into early preventive intervention programs, particularly programs designed to foster children’s early social skills including sharing behavior and prosocial decision-making (Schonert-Reichl et al., 2012). By providing mindful parenting programs early on, it may improve not only the child’s ability to navigate difficulty but also the parent’s ability to regulate their own emotion, thereby embodying the attributes that can then be taught to the child.

## Data Availability

The datasets generated for this study are available on request to the corresponding author.

## Author Contributions

KW and LH contributed to the writing of the manuscript, interpretation of results, and review of the final version of the manuscript. TS contributed to the design of study, data collection, and review of the final version of the manuscript. CT contributed to the review of the earlier versions of the manuscript, data analyses, and review of the final version of the manuscript. TH contributed to the literature search, data analyses, construction of tables, and review of the final version of the manuscript. MZ contributed to funding of the study, study design, interpretation of results, and review of the final version of the manuscript. MH contributed to the study design, data collection, data analyses, interpretation of results, and review of the earlier and final versions of the manuscript.

### Conflict of Interest Statement

The authors declare that the research was conducted in the absence of any commercial or financial relationships that could be construed as a potential conflict of interest.

## Supplementary Material

The Supplementary Material for this article can be found online at: https://www.frontiersin.org/articles/10.3389/fpsyg.2019.00550/full#supplementary-material
