# Supplementary material for: The Role of Mindful Parenting in Individual and Social Decision-Making in Children
Source: Front Psychol. 2019 Mar 20;10:550. doi: 10.3389/fpsyg.2019.00550 (PMC6435956; doi:10.3389/fpsyg.2019.00550)
Supplement: Supplementary file 1 [file Data_Sheet_1.PDF]

Observation: Choice-related Stress

Name observer:

Participant number:

Gender of the child:

<u>TIME:</u>

How long did the child took to pick a present (from the time the box was opened till the time the box was closed again):

Time: ___________ seconds*

* Use the stopwatch!

<u>SCORING – Subscales:</u>

- Stress & Tension:

0= No sign of tension, child is completely relaxed.
1= Child seems somewhat tense (e.g., wiggles in chair)
2= Child seems moderately tense (e.g., fingers in mouth, unease, slightly frowning)
3= Child is clearly tense (e.g., frowning, tense posture)
4= Child is very tense/stressed (e.g., very unease, grim face, child voices being tense/having a hard time, red in the face)

- Doubt:

0= No doubt at all (takes what he/she wants without doubt)
1= Child has little doubt (searches through toys, but picks without difficulty)
2= Child has moderate doubt (inspects and considers some toys, picks with some difficulty)
3= Child is clearly in doubt (inspects and considers several toys, picks toys and puts them back again)
4= Child is highly doubting (inspects and considers many toys or extreme doubt between several (or two) toys, recurring decisions, child voices difficulty, seems afraid of closing the box)

- Confirmation seeking:

0= Child does not ask for help or looks like he/she needs help.
1= Child slightly asks for help without talking (e.g., sometimes looks at test leader and/or thinks out loud)
2= Child asks for help without talking (e.g., looks at test leader and/or thinks out loud)
3= Child carefully asks for help directly (above indirect signs and indirect questions, such as asking carefully what test leader likes)
4= Child strongly asks for help directly (above indirect signs and direct questions, like asking what the test leader would pick or asking to help making a choice).

SCORING - COMMENTS:
